# Why Do Child-Directed Interactions Support Imitative Learning in Young Children?

**DOI:** 10.1371/journal.pone.0110891

**Published:** 2014-10-21

**Authors:** Laura Shneidman, Roisleen Todd, Amanda Woodward

**Affiliations:** University of Chicago, Chicago, Illinois, United States of America; Max Planck Institute for Human Cognitive and Brain Sciences, Germany

## Abstract

Child-directed cues support imitation of novel actions at 18 months, but not at two years of age. The current studies explore the mechanisms that underlie the propensity that children have to copy others at 18 months, and how the value of child-directed communication changes over development. We ask if attentional allocation accounts for children's failure to imitate observed actions at 18 months, and their success at two years of age, and we explore the informational value child-directed contexts may provide across ontogeny. Eighteen-month-old (Study 1) and two-year-old (Study 2) children viewed causally non-obvious actions performed by child-directed (Study 1 & 2), observed (Study 1 & 2), or non-interactive (Study 2) actors, and their visual attention and imitative behaviors were assessed. Results demonstrated that child-directed contexts supported imitative learning for 18-month-old children, independent of their effects on proximal attention. However, by two years of age, neither directness nor communication between social partners was a necessary condition for supporting social imitation. These findings suggest that developmental changes in children's propensity to extract information from observation cannot be accounted for by changes in children's interpretation of what counts as child-directed information, and are likely not due to changes in how children allocate attention to observed events.

## Introduction

Recent findings have highlighted the important role that child-directed teaching contexts play in supporting action imitation. Between 15 and 18 months, young children are more likely to faithfully imitate an individual's actions when they are directly addressed than when they simply observe her actions [1]–[5]. For example, 18-month-old children who watch a person using her head to turn on a light, or her elbow to activate a switch, are more likely to imitate the particular manner of these actions when they are directly engaged than when they view a non-communicative individual [3]. Yet, by the time children are two years of age, child-directed interactions are no longer unique in their ability to support action imitation; two-year-olds are as likely to faithfully imitate novel actions after viewing socially aloof actors as they are after viewing actors who directly engage them [3], [4] and four-year-old children demonstrate equal and robust imitative behaviors when directly taught, when observing a third-party interaction, and when observing a solitary actor [6].

Because faithful imitation of others is critical for the transmission of culturally specified knowledge, it is important to understand the mechanisms that underlie children's propensity to copy others, and how these processes change over ontogeny. In this paper we explore the particular properties of child-directed demonstrations that could matter for supporting action imitation at 18 months, and how the value of child-directed communication might change between 18 months and two years of age. We first ask if children's attentional allocation can account for their failure to learn from observation at 18 months, and their success at two years of age. We next explore if child-directed contexts facilitate learning at 18 months because they contain information that is explicitly marked as being for the child, or because they more generally mark information as worthy of communication. Finally, we consider if two-year-olds' success at learning from observation stems from an increased propensity to imitate observed actors, or from changes in children's interpretation of what counts as being directed to them. Thus, the goal of the current studies is to investigate in greater detail why child-directed contexts matter at 18 months, and how the informational value of these contexts may change over development.

### Is child-directed input important only because it focuses children's attention to relevant pieces of action demonstrations?

Why do 18-month-old children imitate more robustly in child-directed as compared to observational contexts? One possibility is that child-directed contexts do no more than facilitate children's ability to attend to the relevant parts of an action sequence. Indeed, visual salience has been shown to be a mediating factor in determining what actions children imitate [7], and ostensive, social cues can increase this salience [8]–[16]. For example, directed eye contact and child-directed speech attract infants' attention (e.g., [10]–[13]) and infants and young children are more likely to follow an adult's gaze, or gesture, to a referent object following social-ostensive, as compared to non-communicative, cuing [14]–[16]. In contrast, learning from observation requires that children exert independent attentional control in order to glean information from others. Thus, 15- to 18-month-olds' differential imitation of actions in child-directed versus observed contexts could be due to a relative lack of visual attention to the relevant aspects of the non-ostensive event. By two years of age, children may have developed the necessary independent attentional control to learn from observation. This possibility could be assessed by comparing children's attention allocation within child-directed and observed contexts. If 18-month-old children display differing patterns of attention in these situations, and two-year-olds do not, it would support the possibility that 18-month-olds fail to imitate observed actors because they fail to attend to them in effective ways.

A few studies have considered children's attention to child-directed and observed actors, and have found no differences in attentional allocation (e.g., [2], [5]). However, these studies used gross measures of attention (e.g. whether children attended equally overall to the learning contexts), leaving open the question of whether there are more fine-grained differences in how children attend to these situations. If children monitor the specific aspects of these situations in different ways, it could account for the imitation differences found in these studies. Indeed, prior research has found that in the context of learning a new word, 20-month-old children demonstrate equal overall attention to child-directed and observed situations, but differ in their fine-grained attention to these contexts; when overhearing, children deploy relatively more visual attention to the people in the interaction, and less attention to a referent object. Importantly, these differences relate to individual variation in the ability to learn new words from observation [17]. In the current studies we consider the possibility that children's fine-grained attention similarly differs in action observation.

### Is child-directed input important because it marks information as intended for the child?

If children evidence selective learning in child-directed contexts even when the attentional effects of these interactions are controlled for, then this would indicate that child-directed contexts carry informational value beyond the way they focus attention in the moment. What is this value? One possibility is that children are responding to the fact that information is marked as being intended for them. For example, Csibra and Gergely [18]–[20] have posited that child-directed contexts relate to learning through an innately specified response to directed cuing, like child-directed speech or gesture. This response is argued to trigger in the child the assumption that the information conveyed is culturally important and generalizable. Other theorists [21]–[27] propose that child-directed interactions provide the child with a shared focus of attention with another individual. This mutuality facilitates the child's understanding of the other individual's intentions (because they closely match the child's intentions) and thereby supports social learning.

Each of these theories would predict that children should learn more robustly from child-directed interactions, where information is marked as intended for them, as compared to observed teaching interactions, where information is marked as intended for someone else. However, an alternative possibility is that child-directed contexts function more broadly by informing children's pragmatic reasoning about the communicative context. Information that is explicitly taught, either to the child, or to another individual, is marked as intended for someone, and, thus, worthy of communication. Direct teaching may relate to imitation because children reason that communicated information is in some way relevant to the current situation (see [28] for a similar explanation for the relation between child-directed input and learning for preschool children).

In order to explore these alternative possibilities, it is necessary to know if children imitate more robustly following a child-directed teaching context than from a situation where they observe a social and pedagogical interaction. However, most studies that have considered children's action learning from child-directed or observed actors have contrasted a child-directed teaching interaction with a demonstration where an actor spoke to no one (e.g., [1], [2], [4], [5]). A few recent studies ([3], [29]) have included a condition where children watched a 3^rd^ party teaching interaction. In each of these studies the researchers found no significant differences in imitation rates between this condition and a child-directed condition; however, trends in the data of [3] suggested that children may have more robustly imitated when directly addressed, making it difficult to draw strong conclusions from these findings. Further, because these studies did not include a baseline measure of imitation, it is unclear whether children showed differential learning across child-directed and observational conditions. In Study 1, we consider if children show evidence of learning in each of these conditions, and whether they learn more robustly when directly addressed than when observing a teaching interaction. If they do imitate more robustly in child-directed contexts, it would indicate that directedness, and not just general communicative marking, is critical for supporting imitative behaviors.

### How does the informational value of child-directed input change over development?

While 18-month-old children are more likely to imitate causally ambiguous actions after being engaged in child-directed interaction as compared to observing these actions, 24-month-olds are equally likely to imitate actions following child-directed and observational events ([3], [4]). Some theorists have argued that this reflects increases in children's ability to extract relevant information from observational and non-pedagogical contexts (e.g., [3]). However, another possibility is that these changes instead reflect changes in what children interpret as directed to them. Indeed, in the observation condition of one study demonstrating robust imitation from observed contexts at two years of age [4], a second experimenter talked to the child and directed his or her attention to the ongoing demonstration, raising the possibility that older children were broadly interpreting the demonstration as meant for them. Likewise, in a second study [3] a non-engaging social actor spoke to herself about the actions she was performing. Since there was no clear conversational recipient in the room, children may have been interpreting the situation as a pedagogical interaction meant for them. Thus, in order to fully consider the possibility that children's reliance on child-directed contexts decreases over development, it is necessary to test older children's imitation of actors whose communicative intentions are less ambiguous. In Study 2 we assess two-year-old children's imitation following the observation of a person who performs actions while ignoring the child and talking on a phone about topics unrelated to her actions. If children show robust action imitation in this condition, it would provide stronger evidence that by two years of age, children imitate others' actions even when provided with no pedagogical cuing.

In sum, while previous research has demonstrated that child-directed interactions support the imitation of causally opaque actions at 18 months, but not at two years of age, the goal of the current studies is to investigate in greater detail why these contexts matter at 18 months, and how their importance changes over development. In Study 1, we examine the idea that child-directed contexts provide informational value to 18-month-old children beyond the way that these interactions focus attention in the moment, and we consider whether children's imitation reflects a response to information that is marked as being intended for them, or to the broader communicative value that pedagogical interactions provide. In Study 2, we consider why children's reliance on directed cues decreases over development, including a condition where an actor's communicative goals are unambiguously not meant for the child, in order to assess if prior findings could be accounted for by children's changing interpretation of what is meant for them. We then consider how our findings fit into current theoretical models of early learning.

## Study 1

### Method

#### Ethics Statement

**Figure 1:**
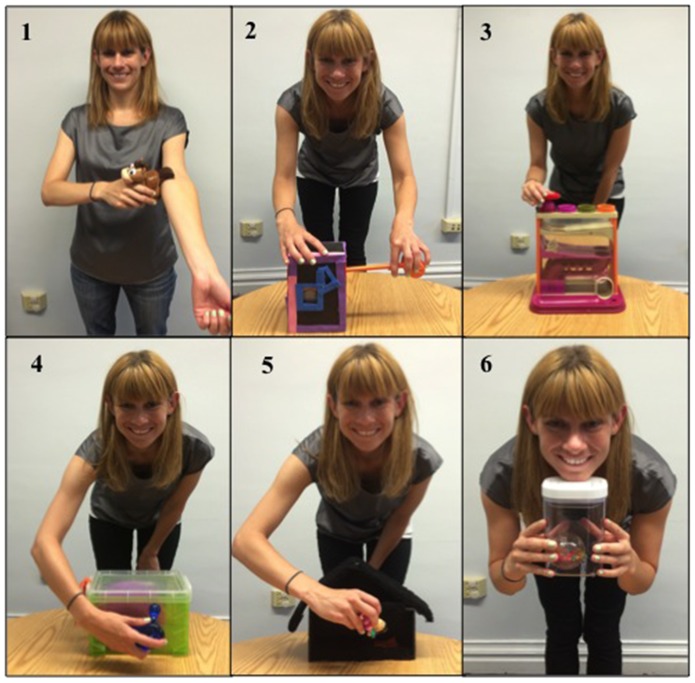
Objects demonstrated in Study 1 and 2.

The Institutional Review Board at the University of Chicago approved the protocol for both Study 1 and Study 2, and children's parents or legal guardians provided written consent prior to children's participation in the experimental protocol. The individual featured in Figure 1 has given written informed consent (as outlined in PLOS consent form) to publish these images.

#### Participants

Thirty-two full-term children from the greater Chicago area participated (Mean age: 18-months; Range: 17.5–18.5 months), sixteen children (8 males, 8 females) in the *Child-directed* condition and sixteen children (8 males, 8 females) in the *Observed* condition. Five additional children participated in the experimental procedure but were excluded from the final sample due to parental interference on more than two test trials (2), fussiness (1), or experimental error (2).

#### Materials

Test items consisted of 6 toys designed to have one functional action that could be performed in a causally opaque manner. Objects included: 1) a toy horse with a hidden button in the tail, which produced a sound when pressed on the demonstrator's upper arm 2) a box that lit up when a plastic tool was pressed on a button 3) a tower that the demonstrator pushed a ball down using a plastic ring 4) a box that was opened with a tab after a block was first knocked against it 5) a cardboard house where a doll was pressed on a patch to activate a sound and 6) a box with a push button lid that was opened with the demonstrator's chin (see Figure 1).

#### Procedure

The child sat on his or her parent's lap in front of a large table. A second smaller table was positioned approximately 7 feet away from where the child was sitting and within his or her visual range. The procedure consisted of two baseline, demonstration, and test phases. All children were presented with all 6 toys during the baseline and test phases (order of object presentation was randomized by an online list generator [30]).

#### Baseline phase

During each baseline phase, an experimenter (the host) presented the child, sequentially, with three test items, saying, “What does this do?” The child then had the opportunity to explore each object for 30 seconds. Following this, the host left the room and two other experimenters (the demonstrator and the conversational recipient) entered the room to begin the demonstration phase.

#### Demonstration phase

In the *Child-directed* condition, both the demonstrator and the conversational recipient made eye contact with the child and each experimenter directed several utterances to the child, in a child-directed speech register, saying things like: “Did you come to play with me today? I'm so glad because I love to share my toys.” Following this, the demonstrator picked up each of the toys in sequence, placed it on the small table, and, while making eye contact with the child, demonstrated how to use the object twice while saying, “Let's see what this thing does.” The *Observed* condition was the same as the Child-directed condition, and included the same phrasing, except instead of addressing the child, the demonstrator and the conversational recipient only spoke to one another. They used a child-directed speech register in order to match the potential attentional salience of the Child-directed condition, but never addressed or made eye contact with the child. After the demonstration phase, both experimenters left the room, and the host returned to begin the test phase.

#### Test phase

During the test phase, the host (who was blind to demonstration condition) once again presented each object to the child and asked, “What does this thing do?” The child had 30 seconds to interact with the toy. Following this, the host initiated the second baseline phase with the remaining three test items, and the entire phase sequence was repeated.

### Measures

#### Visual attention

Children's visual attention during the demonstration phase was coded offline using a digital video coding program [31] for the location and duration of children's visual attention. Attention was classified as directed toward the demonstrator, the conversational recipient, the target objects, or another location. To assess reliability, a second independent assistant, blind to study aims and hypothesis, coded 25% of the participants. Cronbach's Alphas revealed high agreement between the two coders' assessments for the proportion of time children attended to the target object (α = .96), the demonstrator (α = .93), the recipient (α = .95), and the other location (α = .87).

#### Imitation score

Children were scored based on the proportion of objects they operated in a manner consistent with the demonstration at baseline and at test (e.g., they used their upper arm to activate the horse toy, or the tool to operate the light box) by a coder blind to condition. Children were given credit for performing the manner imitation whether or not their actions were effective (e.g. they pressed the horse toy on their upper arm but the toy did not sound). Parental interference occurred in a total of three trials across children in the Child-directed condition. These trials were dropped from the analysis and children received a score based on the remaining items. A second independent assistant, blind to study aims, hypothesis, and condition, coded 25% of the participants, with the coders agreeing on 93% of the total behavioral scores.

### Results

#### Attention to training

**Figure 2:**
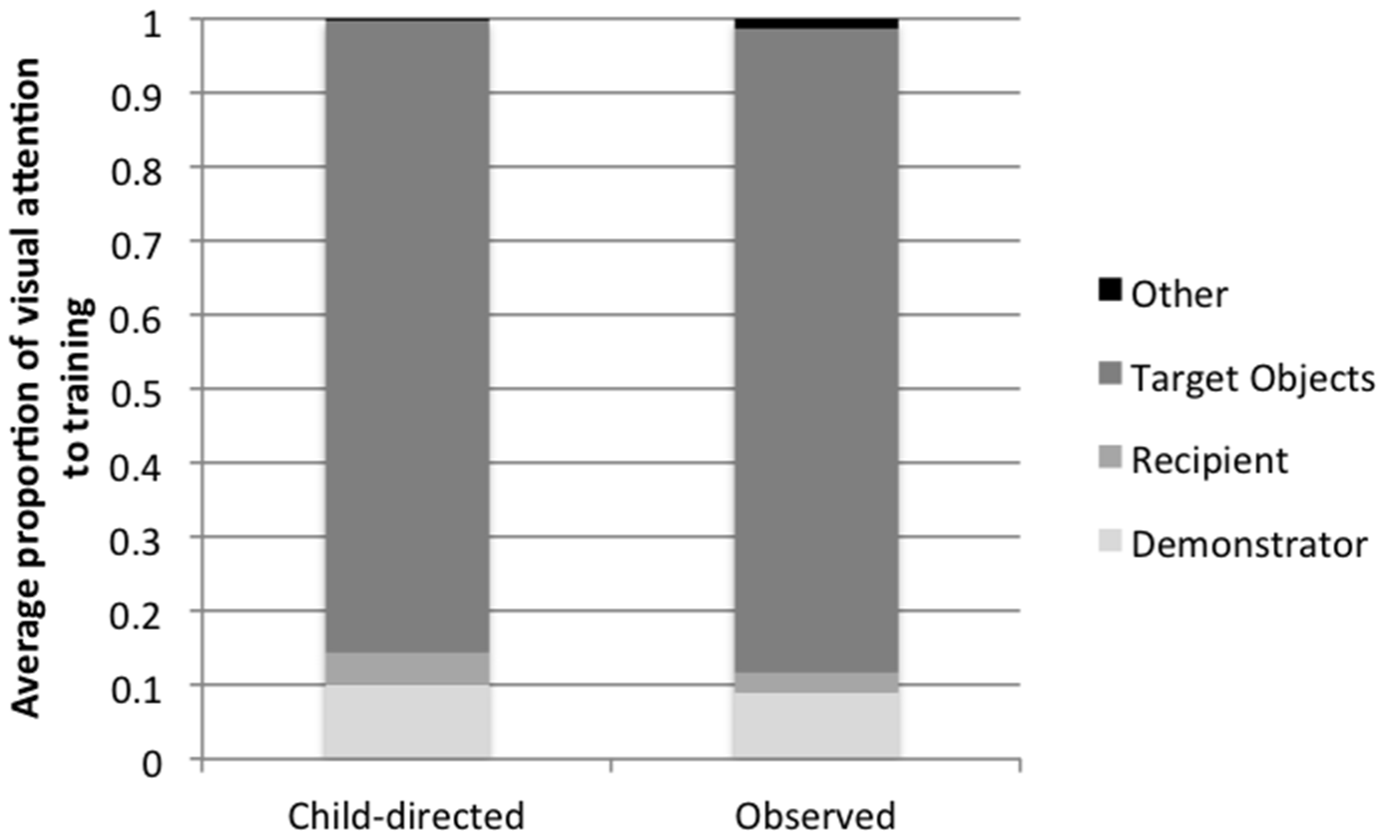
Average proportion of visual attention to elements of the training interaction at 18 months of age (Study 1).

We assessed children's attention to training by considering the proportion of time they looked to the combined relevant elements of the training event (the demonstrator, the conversational recipient, and the target object) relevant to the total training time (see Figure 2). Preliminary analyses revealed no significant effects of children's gender or age on any aspect of attentional allocation so subsequent analyses collapsed across age and sex. Children in both the Child-directed and the Observed conditions were highly attentive to the training events, and there was no difference across condition in the proportion of time they attended to training, *t*(30) = 1.50, *p* = .15. There were also no significant differences in children's attention to the independent elements of the training condition. Children in the two conditions did not differ in their attention to the demonstrator, *t*(30) = .47, *p* = .65; the conversational recipient, *t*(30) = 1.43, *p* = .16; or the target objects, *t*(30) = .61, *p* = .55.

#### Imitation at baseline and test

**Figure 3:**
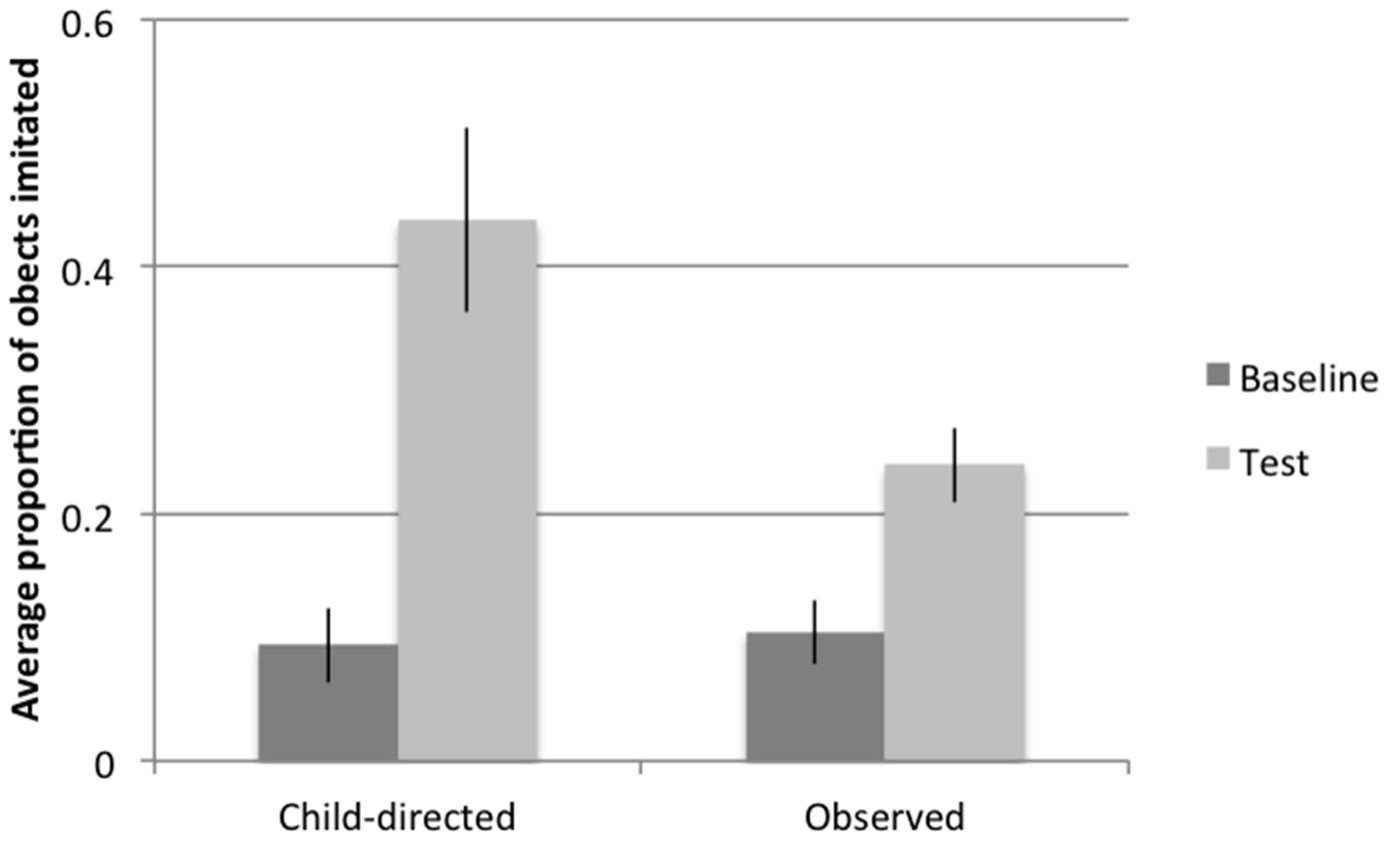
Proportion of actions imitated at baseline and test at 18 months (Study 1).

We next considered children's imitation rates across the two experimental conditions. Preliminary analyses revealed no significant effects of gender or age on imitation, so subsequent analyses collapsed across these measures. A repeated measures ANOVA was conducted on the proportion of actions imitated in the manner demonstrated with trial-type (baseline/test) as a within-subjects-measure and condition (direct/observed) as between-subjects-measures. Results revealed a main effect of trial-type, *F*(1,30) = 27.0, *p*<.001, indicating that children performed more actions at test than at baseline, and of condition, *F*(1,30) = 4.6, *p*<.05, indicating that children in the Child-directed condition performed more actions than children in the Observed condition. There was also a significant trial-type by condition interaction, *F*(1,30) = 5.1, *p*<.05, indicating that children in the Child-directed condition showed greater increases in imitation from baseline to test as compared to children in the Observed condition. Paired comparisons revealed that both children in the Child-directed condition, *t*(15) = 3.90, *p*<.01; and in the Observed condition, *t*(15) = 4.96, *p*<.001 performed significantly more actions at test than at baseline (see Figure 3).

### Discussion

In Study 1 we considered two questions. First, we asked if findings that children imitate more robustly in child-directed as compared to observed situations could be wholly accounted for by differences in children's visual attention. Our results suggest that they cannot. Children allocated their visual attention in very similar ways in the Child-directed and Observed conditions, but children in the Child-directed condition were more likely to imitate the actor's actions, and showed greater increases in imitation rates as compared to children in the Observed condition. Thus, while child-directed interactions may well help focus children's attention in busy social contexts, our results show they must carry informational value beyond this.

We also considered the question of whether child-directed contexts support imitative behaviors because information is marked as intended for the child, or because information is more generally marked as important for communication. We found that children learned more robustly from the child-directed than the observed context, even when the observed demonstration included social and pedagogical information. Thus, directness, and not just general communicative marking, is important for supporting imitative behaviors. However, it is also important to note that children in both the Child-directed and Observed conditions demonstrated learning from baseline to test, suggesting that while direct cuing does support young children's ability to imitate the actions of others, it is not a necessary condition for informing this learning.

Thus, the findings of Study 1 indicate that child-directed interactions do provide important informational value for 18-month-old children. In Study 2, we consider the relation between child-directed interaction and imitative learning for two-year-old children, including a condition where an actor's actions are unambiguously not meant for the child. We compare children's performance in this context to the same two conditions utilized in Study 1. Once again, we explore children's fine-grained attention to each learning context in order to consider the possibility that differences in imitative learning are due to differences in how the modeled actions drive children's attention in the moment. Thus, in Study 2 we are able to test differences in children's imitation to interactive and non-interactive actors at two years, and, by comparing our results to Study 1, we can evaluate potential differences in imitation to child-directed and observed teaching interactions across development.

## Study 2

### Method

#### Participants

Forty-eight full-term children from the greater Chicago area participated (Mean age: 24.9 months; Range: 24.1–26.0 months); sixteen children (8 males, 8 females) in the *Child-directed* condition, sixteen children (8 males, 8 females) in the *Observed* condition, and sixteen children (8 males, 8 females) in the *Non-interactive* condition. Seven additional children participated in the experimental procedure but were excluded from the final sample due to parental interference on more than two test trials (2), fussiness (2), experimental error (1), or because parents did not allow videotaping (2).

#### Procedure

The procedure for children in the Child-directed and Observed conditions was identical to the one described in Study 1. The baseline and test phases of the Non-interactive condition were also identical to Study 1; however, instead of leaving the room, the host turned her attention to a book during the test phase (in order to equate for the total number of individuals present in the other conditions during the demonstration). Then, the demonstrator entered the room, alone, and performed each of the actions described in Study 1 while talking on a phone, using an adult-directed speech register and saying things that were not related to the action sequence she was performing (e.g. “Did you go to the store today? I'm so glad because we needed more of that.”). The demonstrator made no eye contact with either the child or the host. Length of utterance and timing of the performed actions were matched to the Child-directed and Observed conditions.

### Measures

#### Visual attention

Children's visual attention during the demonstration phase was coded offline as described in Study 1 for attention to the demonstrator, the recipient/host, the target object, or another location. One subject in the Child-directed condition was not included in the analysis because her face was not visible in the video recording. To assess reliability, a second independent assistant, blind to study aims and hypothesis, coded 25% of the participants. Cronbach's Alphas revealed high agreement between the two coders' assessments for the proportion of time children attended to the target object (α = .92), the demonstrator (α = .93), the recipient/host (α = .99), and the other location (α = .87).

#### Imitation score

Children were scored based on the proportion of objects they operated in a manner consistent with the demonstration at baseline and at test. Six trials were eliminated from the analysis because of parental interference (three in the Child-directed condition, and three in the Observed-condition). A second independent assistant, blind to study aims, hypothesis, and condition, coded 25% of the participants, with the coders agreeing on 90% of the total behavioral scores.

### Results

#### Attention to training

**Figure 4:**
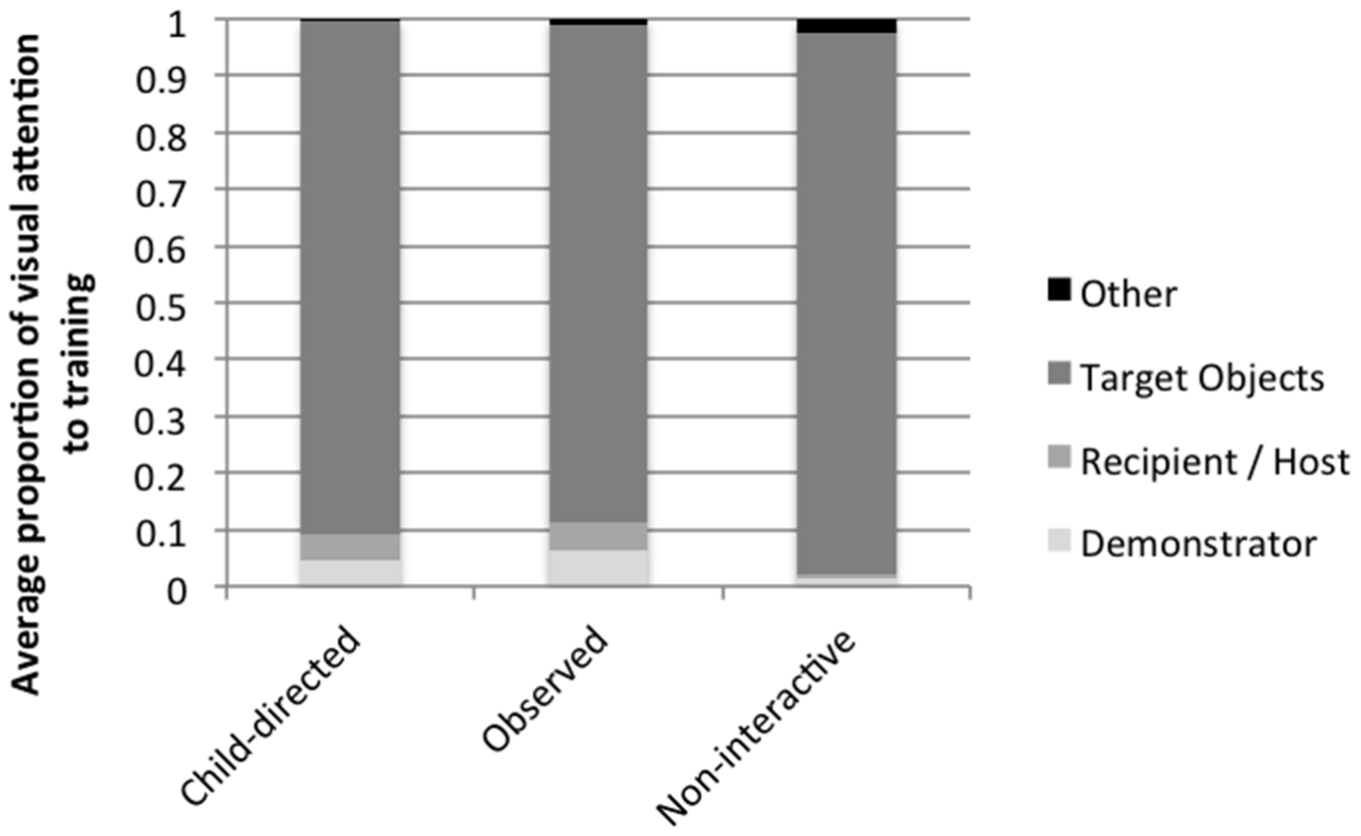
Average proportion of visual attention to the elements of training interaction at 25 months of age (Study 2).

Preliminary analyses revealed no significant effects of child gender on attention to the training or imitation, so subsequent analyses collapsed across these factors. There were differences across conditions in the overall average proportion of time children allocated to the training event, *F*(2,44) = 3.4, *p*<.05, as well as to the individual elements of the interaction, including: the target objects, *F*(2,44) = 5.1, *p*<.05; the demonstrator, *F*(2,44) = 8.9, *p*<.01; and the conversational recipient (host in the Non-interactive condition), *F*(2,44) = 3.9, *p*<.05 (see Figure 4).

Post-hoc tests revealed no differences in how children allocated attention between the Child-directed and Observed conditions. However, children in the Non-interactive condition allocated relatively less overall attention to the interaction than children in the Child-directed condition (*t*[29] = 2.2, *p*<.05). Additionally, these children allocated relatively more visual attention to the target object than children in the Child-directed (*t*[30] = 2.3, *p*<.05) or Observed (*t*[30] = 3.2, *p*<.01) conditions, and relatively less attention to the demonstrator as compared to children in the Child-directed (*t*[30] = −4.0, *p*<.001) and Observed (*t*[30] = −3.7, *p*<.01) conditions. Children in the Child-directed (*t*[29] = 3.4, *p*<.01) and Observed (*t*[30] = 2.4, *p*<.05) conditions allocated more attention to the second person in the room (the conversational recipient) than children in the Non-interactive condition (the host).

We next assessed whether children's attention differed between 18 and 25 months by comparing children's attention in Study 2 to the attention measures from Study 1 in the Child-directed and Observed conditions. Results demonstrated that, across conditions, 18-month-old children allocated relatively more visual attention to the demonstrator during training than did 25-month-old children, *F*(1,59) = 10.5, *p*<.01. There were no other significant differences in children's attentional allocation across the two studies.

#### Imitation at baseline and test

**Figure 5:**
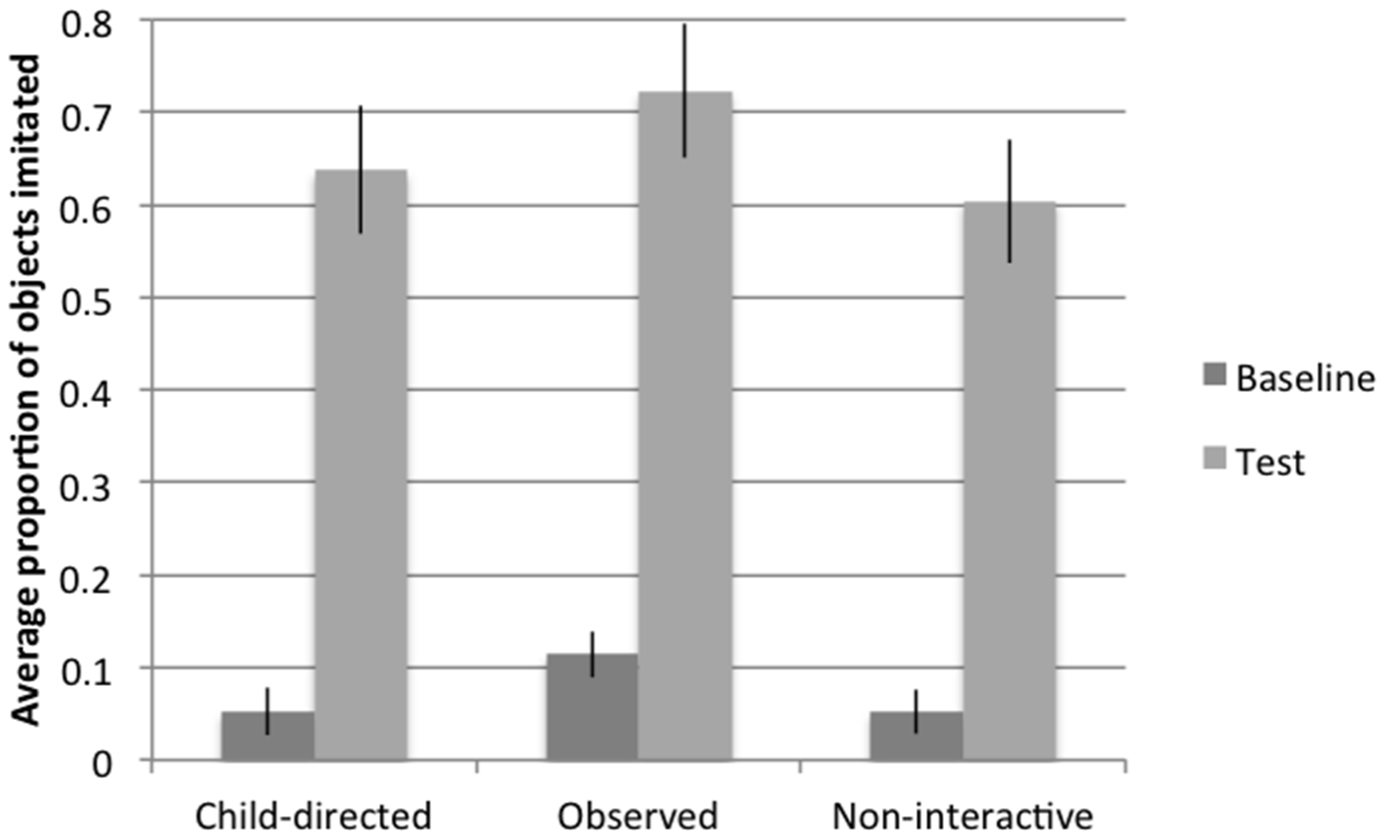
Proportion of actions imitated at baseline and test at 25 months (Study 2).

Despite differences in attentional allocation, two-year-old children showed similar imitation rates across the three conditions. A repeated measures ANOVA was conducted on the proportion of actions imitated in the demonstrated manner with trial-type (baseline/test) as a within-subjects-measure, and condition (Child-directed/Observed/Non-interactive) as a between-subjects-measure. Results revealed a main effect of trial type, *F*(1,45) = 176.1, *p*<.001, indicating that across conditions children showed significant increases in the proportion of actions imitated from baseline to test. There was no main effect of condition, *F*(2,45) = 1.8, *p* = .19, and no trial type by condition interaction, *F*(2,45) = .14, *p* = .88. Thus, children in all three conditions performed more actions at test than at baseline, and there were no differences in the change between baseline and test imitation in the conditions (see Figure 5).

### Discussion

These findings indicate that by two years of age, children robustly imitate the actions of others regardless of whether they are directly taught, they observe a teaching interaction, or they observe an actor with no intention to teach. The finding that children learn actions from even a non-interactive, non-pedagogical actor goes against the hypothesis that, by two years, children have simply broadened their interpretation of what counts as child-directed information. Instead, the reliance on child-directed contexts to inform imitative behavior seems to disappear between 18 and 25 months.

Because children demonstrated robust imitative behavior in all conditions, differences in attentional distribution cannot account for children's learning patterns. Nevertheless, we found that children in the Non-interactive condition allocated relatively less attention to the overall interaction, and to the experimenters, and relatively more attention to the target object than did children in the Child-directed and Observed conditions. These findings are likely due to the experimenters, and the situation, being less interesting to watch in the Non-interactive condition than in the other two conditions. The demonstrator in the Non-interactive condition used an adult-directed speech register and made no social eye contact, and the host had a non-active social role. We also found that, across conditions, 18-month-olds attended more to the demonstrator than 25-month-old children did during training. This may reflect changes in the way children generally allocate attention to ongoing events between 18 months and two years. However, because we found no difference within each age group in attention to the Child-directed and Observed conditions, these developmental changes are unlikely to drive changes in children's ability to extract information from observed interactions.

## General Discussion

Our goals were to consider the mechanism through which child-directed communication relates to imitative learning by examining the informational value that child-directed teaching contexts provide, and by considering changes in this value over early development. Our findings add to the current literature by demonstrating that directness is an important cue for supporting social imitation at 18 months, and that this is not due to differential attention that children deploy to child-directed and observed contexts, nor to the broader pragmatic information that teaching interactions may provide. Our findings also demonstrate that developmental changes in children's propensity to extract information from observational contexts cannot be accounted for by changes in children's interpretation of what counts as child-directed information, and are likely not due to changes in how children allocate attention to observed contexts. Instead, by the time children are two years of age, they robustly copy other's actions, and neither directedness nor communication between social partners is a necessary condition for supporting this imitation.

How do these results fit in with current accounts of imitative learning? One theory is that child-directed teaching interactions provide automatic, a priori informational value regarding which aspects of another's actions are most important to learn and generalize [18]–[20]. Under this model, when children observe an action sequence with non-obvious causal properties, they have no way of knowing which pieces of the sequence should be replicated. However, it is argued that children automatically assume that information accompanied with communicative cues such as eye contact and child-directed speech is culturally important and generalizable, and thus should be learned. While this account is consistent with our data from 18-month-olds, we believe that the developmental changes observed from 18 to 25 months make it unlikely that children's behavior is due to this kind of fixed reaction to a modular learning system. By the time children are two years of age, they imitate cognitively opaque actions even when there is no social or pedagogical cuing. If children's responses at 18 months reflect a response to a modular and automatic system, it is puzzling why responses to such a system would change so drastically in six short months.

Thus, we take our findings to be more compatible with, but broader than, two recent theoretical proposals. One account [21]–[27] proposes that child-directed interactions are critical for early learning because they provide the child with a shared focus of attention with another individual. This mutuality facilitates the child's understanding of the other individual's intentions (because they closely match the child's intentions) and thereby supports social learning. This account assumes that initially, children are unlikely to learn from 3^rd^ party interaction because children have difficulty understanding the intentions of others in the absence of mutual focus. This reliance on episodes of child-directed interaction may thus decrease as social cognitive abilities develop (e.g., [24], [25]). For example, Moore [25] has hypothesized that the ability to learn from observation depends on children's developing ability to take on the perspectives of others. As children develop, they become better able to take on the perspectives of other individuals (even when they differ from their own), and thus become more likely to learn from social interactions that do not involve ostensive engagement.

Our results are consistent with this proposal; we found that that, at 18 months, the directness of a pedagogical interaction was important for supporting action imitation, but that by two years of age, children no longer showed differential imitation to directed as compared to observed actors. Even so, while child-directed interactions could strengthen children's understanding of other's intentions, a broader look at the literature leaves reason to doubt strong claims that child-directed interactions are ever critical for supporting this understanding. Before infants are old enough to engage in episodes of mutual focus with others, they view other's actions as structured by intentions, suggesting that mutual interaction is not a necessary condition for understanding other's actions (e.g., [32]). Furthermore, children demonstrate a robust ability to learn new words from observation at 18 months of age, the earliest age at which they have been tested ([33], [34]). Under an intentional understanding account, it is unclear why learning actions, but not learning words, should rely on mutual engagement. In both contexts, children presumably have to understand the actor's intentions in order to demonstrate learning.

A second theoretical perspective proposes that developmental differences in imitation rates to observed actors reflect changes in children's social goals across development [4]. Nielsen [4] has suggested that at 18 months, children who are directly engaged by an experimenter could be motivated to sustain this interaction, and thus imitate actions she performs, even when they seem unrelated to any goal. In contrast, children may have no such motivation when they view a socially aloof model. By two years of age, children may have the desire both to sustain social interaction, and to initiate an interaction with someone who is not engaging them. Thus, they may be motivated to imitate causally opaque actions even in the absence of mutual focus.

Our findings are not consistent with the specific prediction that children's motivation to engage non-interactive partners changes with development; we found that at two years of age, children imitated a socially disengaged model, even though she was not present in the room when the child had the opportunity to interact with the test objects. Thus, it seems unlikely that children were trying to initiate a social interaction with this person. However, a broader conceptualization of social motivation could explain the changes we observed between 18 and 25 months. Young children could have changing ideas about what child-directed and observed contexts mean in terms of what they should, can, or would like to do. Indeed, there is ample evidence that, in other contexts, social motivation and affiliation do affect children's imitation rates (see [35]). For example, 18-month-old children are more likely to imitate actors when they have prior familiarity with the individual [29], and their imitation is moderated by the emotional affect visually present others have shown in response to demonstrated actions [36]; toddlers and preschool children are more likely to imitate actors who speak the child's native language as opposed to actors who do not ([37], [38]); and in toddlers imitation is moderated by whether the actor is on video, or is a live social interlocutor [38].

Thus, the information children take from child-directed interactions could depend on their social conceptual understanding, including their understanding of the actor's intentions and the pragmatics of the learning context, as well as their motivation to learn or to reproduce the behaviors they have seen. These possibilities open a number of questions for future research. One question is how children's comprehension of culturally specified actions learned in child-directed and observed contexts may differ from their action production. If child-directed contexts facilitate imitation because they signal to children what they are allowed or expected to do, then one would expect children to perform differently in tests that assess children's comprehension and production of observed actions. Future research might devise a passive measure of action learning that could help to differentiate whether 18-month-old children are less likely to learn novel actions from observed situations, or, alternatively, are learning these actions but choosing not to replicate them when given the chance to interact with the object.

The motivation or propensity to learn from observation may also differ depending on children's early experiences. Children in our sample were growing up in a cultural context where child-directed instruction is commonplace, and, as such, it is possible they had certain expectations about the importance or relevance of information conveyed by child-directed teaching. However, many children live in cultural environments where they receive more observational experience, and children are expected to learn from observing the actions of others (e.g., [39], [40]). These children may have different kinds of motivations or expectations regarding the value child-directed and observed contexts provide. Indeed, prior research suggests that even within-cultural variation in social experience relates to children's ability to learn new words from observation [17]. Future research might consider if similar kinds of variation related to differential action imitation to child-directed and observed actors, given that children in similar communities demonstrate robust imitative behaviors following pedagogical teaching ([41], [42]).

In summary, our results with 18-month-old children suggest that child-directed contexts do provide unique informational value for children, independent of their effects on proximal attention, and on their broader communicative value. However, our findings with two-year-old children leave reason to doubt that this value is a result of an automatic learning system that responds uniquely to child-directed contexts. Instead, imitation from observation may rely on children's developing understanding of others' intentions, or on children's changing pragmatic interpretation of what is relevant, expected, or desirable in child-directed and observed contexts.

## Supporting Information

File S1 **Spreadsheet containing children's imitation score at baseline and test across trials and children's visual attention during demonstration.**
(XLSX)
